# Changes in mental health stigma and well-being: knowledge, attitudes and behavioural intentions among Hong Kong residents between 2021 and 2023

**DOI:** 10.1192/bjo.2025.10865

**Published:** 2025-10-23

**Authors:** Stephanie Ng, Odile Thiang, Young Suk Oh

**Affiliations:** School of English, The University of Hong Kong, Hong Kong, China; School of Nursing, The Hong Kong Polytechnic University, Hong Kong China; Mind HK, Hong Kong, China; Lee Shau Kee School of Business and Administration, The Hong Kong Metropolitan University, Hong Kong, China

**Keywords:** Mental health, mental illness, stigma and discrimination, subjective well-being, Hong Kong

## Abstract

**Background:**

Previous research has demonstrated that the COVID-19 pandemic led to a global increase in mental distress. However, few studies have examined the impact of the pandemic on mental health stigma.

**Aims:**

To investigate changes in measures of mental health stigma, including knowledge, attitudes and behavioural intentions, in 2021 and 2023 in Hong Kong; to examine the mediating role of attitudes on the relationship between knowledge and behavioural intentions; and to explore how disclosure of mental illness contributes to enhanced overall well-being.

**Method:**

Data were collected as part of a larger research project focusing on mental well-being in Hong Kong. A total of 1010 and 1014 participants were surveyed in 2021 and 2023, respectively. The participants were Hong Kong residents aged 18 years and above.

**Results:**

Our findings demonstrate that all measures of mental health stigma showed increases in severity between 2021 and 2023. In addition, our mediation analyses observed both full and partial mediation effects of attitudes on the relationship between knowledge and behavioural intentions. The results also showed that mental illness disclosure was associated with higher well-being; however, despite these benefits, there was a decrease in willingness to disclose in 2023 compared with 2021.

**Conclusions:**

This study highlights the ongoing issue of mental health stigma in Hong Kong. Future mental health programmes and interventions should aim to address various facets of mental health knowledge, including symptom recognition, access to support resources and the deleterious consequences of mental health stigma.

The coronavirus disease (COVID-19) pandemic resulted in an uptick in mental health distress globally.^1^ In addition to the physical health consequences of contracting the virus, the pandemic and the various public health protection measures implemented in its wake led to a vast array of associated well-being consequences, such as bereavement, isolation, income loss, disruption of daily routine and increased domestic violence, which contributed to the development of psychological distress or exacerbated existing mental health struggles.^2,3^ Notably, within the Hong Kong context, strict quarantine measures and isolation protocols resulted in increased stress, anxiety and self-imposed isolation due to fear of infection and contact tracing implications.^4^ These significant mental health implications of the pandemic also led to an increased sense of urgency on the part of the government and local non-governmental organisations with respect to addressing mental-health-related issues in Hong Kong society. This urgency is evidenced by an observed increase in research and intervention efforts directed towards examining and addressing these concerns.^5–8^

Closely related to the observed rise in instances of psychological distress is the issue of mental health stigma. As defined by psychiatrist Graham Thornicroft, mental health stigma consists of three components, namely (a) problems of knowledge (ignorance), (b) problems of attitudes (prejudice), and (c) problems of behaviour (discrimination).^9^ The consequences of mental health stigma are pervasive and significant, affecting the well-being of individuals with psychological distress as well as the communities around them. For instance, mental health stigma can subject individuals with existing psychiatric diagnoses to discriminatory treatment across different realms of their lives (e.g. within their families, healthcare settings and professional contexts), as well as discouraging individuals who are struggling with their mental health from taking preventive measures or reaching out for support.^10,11^

In addition, the existing literature on mental health stigma has highlighted the significant harms of self-stigma; this term describes how individuals with mental health struggles may internalise stigmatising beliefs and attitudes about people struggling with mental health concerns.^12,13^ Research on self-stigma has further demonstrated a negative correlation between levels of self-stigma and the individual’s willingness to disclose their mental health struggles.^14,15^ Crucially, reluctance to disclose one’s struggles has been linked to negative outcomes including decreased self-esteem, poorer quality of interpersonal relationships and delayed help-seeking behaviours.^16,17^

The data on mental health stigma in Hong Kong is limited and has focused primarily on the perceived experience of stigma by individuals with mental illness, rather than on population-wide stigma levels. Previous stigma research in Hong Kong has shown that individuals with mental illness report experiences of significant stigma related to their mental illness and their use of psychiatric support services,^18–20^ which have subsequent negative impacts on their well-being and quality of life, as well as their self-perception and self-stigma. There is an evident gap in the literature with respect to population-wide mental health stigma levels, indicating a need for a study to provide an understanding of the mental health stigma landscape in Hong Kong.

In light of these findings, in the present study, we aimed to examine changes in specific stigma-related outcomes, including mental health knowledge, attitudes, and behavioural intentions, between the years of 2021 and 2023 among the general population of Hong Kong. In addition, we aimed to examine the causal relationships of these changes, specifically, the mediating role of individuals’ attitudes in the effects on their knowledge and behavioural intentions. Notably, the timeframe of the study encompasses the peak of and gradual decrease in pandemic-related concerns and associated public health measures.^2,21^ Considering the relationship between self-stigma and willingness to disclose personal mental health struggles and well-being, we examined how an individual’s disclosure of mental illness contributed to enhancing their overall well-being.

## Method

The current study is a part of a broader research project with the Mind Mental Health Hong Kong Limited (Mind HK; Hong Kong S88 charity number: 91/16471) titled ‘Mental Health in Hong Kong: Assessing Mental Well-Being, Mind HK Programs and Resources, and Mental Health Literacy, Support, and Stigma’. The authors assert that all procedures contributing to this work comply with the ethical standards of the relevant national and institutional committees on human experimentation and with the Helsinki Declaration of 1975, as revised in 2013. All procedures involving human participants and/or patients were approved by Human Research Ethics Committee of The University of Hong Kong (protocol number: EA2006029). The primary objective of the project is to assess mental well-being within the Hong Kong population. Furthermore, it aims to investigate the factors that influence individuals’ mental well-being, such as mental health stigma and availability of resources, and contribute to enhancing mental health support systems and resources in Hong Kong.

### Data collection procedures

The data were collected among Hong Kong residents aged 18 years or above with Cantonese, Mandarin or English proficiency. All authors are current staff at Mind HK, which commissioned a data collection service company, Social Policy Research Limited, to gather the data via telephone surveys. Individuals under the age of 18 years were excluded from this study. A random sampling technique was employed by the service provider for data collection to mitigate any potential selection bias. This method involved generating two sets of phone numbers. The first set consisted of numbers randomly selected from telephone directories, whereas the second list was derived from the first list using the plus-minus one-two technique. After removal of all duplicate numbers, the final sample comprised approximately 70% mobile and 30% landline numbers.

A group of well-trained interviewers from the service provider conducted the phone interviews after obtaining verbal consent from the respondents. The first round of data collection occurred between 23 August and 15 September 2021. Each telephone survey took approximately 20 to 30 min to complete. The respondents were required to complete at least 90% of the questions for the interview to be considered successful. Each number was attempted five times before contact was deemed unsuccessful. A total of 4000 numbers were dialled, with 1987 deemed to be invalid for reasons including being fax or data lines, non-residential lines or non-working lines. Using the remaining 2013 valid numbers, successful interviews were conducted with 1010 respondents, resulting in a response rate of 50.2%.

The second round of data collection took place between 9 and 27 June 2023. It should be noted that the two rounds of data collection represented independent cross-sectional surveys. That is, the samples obtained in 2021 and 2023 were separate, and no individual respondent was tracked across both time points. A total of 3800 numbers were dialled, with 1784 deemed to be invalid for the aforementioned reasons. Among the remaining 2016 valid numbers, successful interviews were conducted with 1014 respondents, resulting in a response rate of 50.3%. The sample size of 1000 or more participants was determined using a conservative estimate of 50%, a common practice when the true population proportion is unknown.^23^ This method provides a cautious and balanced estimation for the sample size. With this sample size, we achieved a 95% confidence level, ensuring that our estimates maintained a maximum sampling error of ±3.1%. By employing random sampling techniques and maintaining this sample size across each round, we could consider the results obtained from the surveys to be reliable and representative of the broader population. This approach thus ensured a robust representation of the target demographic and enhanced the credibility of the findings.

Table 1 provides detailed demographic information corresponding to the data collection year. The rationale for collecting these demographic variables was based on their potential influence on the dependent variables in our study. Previous research has indicated that demographic factors such as gender, age and socioeconomic status can affect these outcomes.^24^ As such, we aimed to reduce potential confounding effects and provide more accurate estimates of the relationships being studied by including gender, age, ethnicity, education level and economic status (henceforth referred to as demographic information) as covariates in our analyses.

**Table 1:** Demographic information of survey participants

Characteristic	2021 (*N* = 1010)			2023 (*N* = 1014)		
	*N*	%	*M* (s.d.)	*N*	%	*M* (s.d.)
Gender	1010			1014		
Male	452	44.8		488	48.1	
Female	558	55.2		526	51.9	
Age, years			44 (15)			47 (16)
Ethnicity						
Chinese	1009	99.9		993	97.9	
Non-Chinese	1	0.1		21	2.1	
Education						
No schooling	6	0.6		8	0.8	
Primary	67	6.6		81	8.0	
Lower secondary	119	11.8		166	16.4	
Upper secondary	414	41.0		293	28.9	
College	151	15.0		180	17.8	
Bachelor’s degree	236	23.4		258	25.4	
Postgraduate	17	1.7		28	2.8	
Economic activity						
Working	664	65.7		606	59.8	
Student	49	4.9		67	6.6	
Homemaker	146	14.5		417	14.5	
Retired	113	11.2		161	15.9	
Unemployed	38	3.8		33	3.3	
Mental illness disclosure						
Yes	62	41.9		57	35.2	
No	86	58.1		105	64.8	

### Measures

The current study employed a series of internationally validated instruments to assess both mental health stigma and overall well-being. Specifically, mental health stigma was measured using the Mental Health Knowledge Schedule (MAKS), the Community Attitudes towards the Mentally Ill (CAMI) scale and the Reported and Intended Behaviour Scale (RIBS). In addition, participants’ overall well-being was evaluated using the World Health Organization Well-Being Index (WHO-5).^22^ All instruments have been validated in both English and Chinese across diverse global contexts, ensuring robust and comparable measurements. The following subsections provide detailed descriptions of each measure.

#### Stigma-related mental health knowledge

Participants’ stigma-related mental health knowledge was assessed using the MAKS.^9^ Specifically, the MAKS scale is divided into two subsections. The first subsection (MAKS-A) consists of six items (e.g. ‘If a friend had a mental health problem, I know how to advise them to get professional help’) measured on a five-point Likert-type scale ranging from 1 (strongly disagree) to 5 (strongly agree), with higher scores indicating lower stigma. The second subsection consists of six conditions (e.g. depression). Each condition is measured on a five-point Likert-type scale ranging from 1 (strongly disagree) to 5 (strongly agree), with scores indicating respondents’ perception of the condition being diagnosable. The MAKS is a widely recognised and reliable tool that has been employed in various global contexts including Germany,^25^ India,^26^ Jordan,^27^ Portugal^28^ and Hong Kong.^29^ Despite the widespread use of the MAKS, there needs to be more consistency in how researchers apply it in their research. For instance, some studies combined all MAKS items for a comprehensive analysis, which allowed the researchers to gain a holistic view of mental health knowledge,^26,30,31^ whereas others focused on the first six items of the MAKS.^24,25,32^ This variation in methodological approaches highlights the flexibility of the MAKS but also points to a lack of standardisation in its application. In response to these differing methodologies, the current study adopted a dual approach, employing both the first six items and the combined MAKS scores for analysis.

#### Attitudes towards individuals with mental illness

The CAMI scale was used to measure participants’ attitudes towards persons with mental illness. The original CAMI scale consists of 40 items;^33^ however, the current study utilised a 12-item CAMI scale.^34^ The 12-item CAMI scale is categorised into two subscales. The Prejudice and Exclusion (CAMI-P/E) subscale consists of six negatively phrased items (e.g. ‘People with mental illness don’t deserve our sympathy’), whereas the Tolerance and Support (CAMI-T/S) subscale comprises six positively phrased items (e.g. ‘Mental illness is an illness like any other’). Both the CAMI-P/E and the CAMI-T/S were measured on a five-point Likert-type scale ranging from 1 (strongly disagree) to 5 (strongly agree). The two subscales have been shown to have acceptable Cronbach’s *α* values (*α* = 0.77–0.84 for CAMI-P/E and *α* = 0.67–0.73 for CAMI-T/S) in previous studies.^24,35^ As such, the 12-item CAMI scale was used in the current study.

#### Intended behaviours towards individuals with mental illness

We used the RIBS^36^ to measure participants’ intentions towards future contact with persons with mental illness. RIBS comprises four items (e.g. ‘In the future, I would be willing to live with someone with a mental health problem’) measured on a five-point Likert-type scale ranging from 1 (strongly unwilling) to 5 (strongly willing), with higher scores indicating better stigma outcomes. The four-item RIBS scale has been shown to have an acceptable Cronbach’s *α* value (*α* = 0.85)^36^ and was thus deemed appropriate for use in the current study.

#### Subjective well-being

The WHO-5 was used to measure participants’ subjective well-being. It is composed of five items (e.g. ‘I have felt cheerful and in good spirits’), which are positively phrased, measured on a six-point Likert-type scale ranging from 0 (at no time) to 5 (all of the time). A total score of 25 indicates maximal well-being, whereas 0 represents an absence of well-being. According to a systematic review study of the WHO-5,^22^ the scale is a valid tool for screening individuals’ subjective well-being.

## Results

Before the analyses were conducted, the data were examined for potential multivariate outliers using Mahalanobis distance and the chi-squared distribution function (*P* < 0.001) in SPSS version 25 for Windows (IBM, Armonk (New York) https://www.ibm.com/products/spss). The screening process identified 95 cases that exceeded the *P* ≤ 0.001 threshold.^37^ Despite this, all results remained consistent whether or not the outliers were included, suggesting that these outliers did not significantly influence the analysis. Therefore, the decision was made to retain all outliers to preserve the integrity of the raw data-sets.

### Comparison of stigma-related mental health knowledge, attitudes and intended behaviours between 2021 and 2023

One-way multivariate analysis of covariance was performed using the general linear model multivariate procedure within SPSS version 25 to assess the change in participants’ knowledge, attitudes and intended behaviours towards persons with mental illness, while controlling for participants’ demographic information, between the years 2021 and 2023. The multivariate test using Wilks’ lambda indicated a significant difference in the combined means of the dependent variables, *Λ* = 0.87, *F*(5, 2013) = 60.18, *P* < 0.001, *η*_p_^2^ = 0.13. Specifically, there were statistical differences in participants’ stigma-related mental health knowledge for both MAKS-A (*F*(1, 2017) = 175.67, *P* < 0.001, *η*_p_^2^ = 0.08) and MAKS (*F*(1, 2017) = 58.54, *P* < 0.001, *η*_p_^2^ = 0.03) and in attitudes towards mental illness for both CAMI-P/E (*F*(1, 2017) = 72.50, *P* < 0.001, *η*_p_^2^ = 0.04) and CAMI-T/S (*F*(1, 2017) = 119.75, *P* < 0.001, *η*_p_^2^ = 0.06), as well as differences in RIBS (intended behaviours towards persons with mental illness; *F*(1, 2017) = 23.26, *P* < 0.001, *η*_p_^2^ = 0.01). In other words, over the 2 years, participants’ stigma-related mental health knowledge, tolerance- and/or support-related attitudes and intended behaviours towards persons with mental illness decreased, whereas prejudice- and/or exclusion-related attitudes increased (Table 2).

**Table 2:** Stigma-related knowledge, attitudes and intended behaviours between 2021 and 2023

Variable	*M* (s.d.) 2021	*M* (s.d.) *2023*	*b*	s.e.	*t*	Bootstrap 95% CI	
						Lower	Upper
MAKS	44.97 (4.05)	43.59 (3.92)	−1.35	0.18	−7.65**	−1.70	−1.00
MAKS-A	23.01 (2.78)	21.45 (2.57)	−1.58	0.12	−13.25**	−1.82	−1.35
CAMI-P/E	17.47 (3.61)	18.86 (3.51)	1.34	0.16	8.52**	1.03	1.64
CAMI-T/S	22.55 (3.20)	21.08 (2.91)	−1.50	0.14	−10.94**	−1.77	−1.23
RIBS	12.75 (3.72)	11.93 (3.38)	−0.77	0.16	−4.82**	−1.08	−0.45

MAKS, Mental Health Knowledge Schedule; MAKS-A, Mental Health Knowledge Schedule, first subscale; CAMI-P/E, Community Attitudes towards the Mentally Ill, Prejudice and Exclusion subscale; CAMI-T/S, Community Attitudes towards the Mentally Ill, Tolerance and Support subscale; RIBS, Reported and Intended Behaviour Scale.

**P < 0.001.

### Individual well-being based on mental health disclosure

An independent samples *t*-test was conducted to assess whether participants’ well-being differed on the basis of their mental health disclosure. The results indicated a statistically significant difference in well-being between participants who disclosed their mental illness (*M* = 10.18, s.d. = 4.49) and those who did not (*M* = 7.16, s.d. = 3.05); *t*(160) = 5.06, *P* < 0.001, *d* = 0.79, 95% CI: [1.84, 4.19]. Specifically, individuals who disclosed their mental illness exhibited higher levels of well-being. Notably, however, willingness to disclose decreased from 41.9% in 2021 to 35.2% in 2023 (Table 1).

### Relationship between stigma-related mental health knowledge, attitudes and intended behaviours

The PROCESS macro for SPSS v.4.2 model 4 was used to explore the relationship between participants’ knowledge (measured by both MAKS-A and MAKS), attitudes and intended behaviours towards persons with mental illness, while controlling for demographic variables. Specifically, data from both the 2021 and 2023 cross-sectional surveys were combined into a single data-set, which enabled examination of the overall relationships between knowledge, attitudes and intended behaviours across the full sample. On the basis of the analyses, all the criteria for conducting a mediation analysis were met.^16^ As shown in Table 3 (MAKS-A → CAMI-T/S → RIBS), the analysis confirmed a positive relationship between MAKS-A and CAMI-T/S (*b* = 0.355, s.e. = 0.024, *P* < 0.001). In addition, it demonstrated a positive effect of CAMI-T/S on RIBS (*b* = 0.380, s.e. = 0.025, *P* < 0.001). Furthermore, the direct effect of MAKS-A on RIBS, when CAMI-T/S was included, was significant (*b* = 0.117, s.e. = 0.028, *P* < 0.001), suggesting potential partial mediation. The indirect effect of MAKS-A on RIBS via CAMI-T/S was significant (*b* = 0.135, s.e. = 0.015, 95% CI: [0.106, 0.165]), indicating that CAMI-T/S serves as a partial mediator in the relationship between MAKS-A and RIBS. In simpler terms, the relationship between MAKS-A and RIBS is not fully explained by CAMI-T/S alone. Other elements, such as personal experiences, social influences, environmental factors and individual beliefs, could also have crucial roles in determining individuals’ behavioural intentions towards those with mental illness.

**Table 3:** Direct, indirect and total effects of six-item stigma-related mental health knowledge on intended behaviours via attitudes towards mental illness

Path	*b* ^a^	s.e.	*t*	Bootstrap 95% CI^b^	
				Lower	Upper
(a) Direct effects					
MAKS-A → CAMI-T/S	0.355	0.024	14.813**	0.308	0.401
CAMI-T/S → RIBS	0.380	0.025	15.427**	0.332	0.429
MAKS-A → RIBS	0.117	0.028	4.184**	0.062	0.172
(b) Indirect effects					
MAKS-A → CAMI-T/S → RIBS	0.135	0.015	–	0.106	0.165

MAKS-A, Mental Health Knowledge Schedule, first subscale; CAMI-T/S, Community Attitudes towards the Mentally Ill, Tolerance and Support subscale; RIBS, Reported and Intended Behaviour Scale.

a. *b* indicates unstandardised coefficients

b. Bootstrap sample size = 5000.

**P < 0.001.

Similarly, as shown in Table 4 (MAKS → CAMI-T/S → RIBS), a positive relationship between MAKS and CAMI-T/S was confirmed (*b* = 0.218, s.e. = 0.017, *P* < 0.001), and CAMI-T/S was also positively related to RIBS (*b* = 0.401, s.e. = 0.025, *P* < 0.001). However, the direct effect of MAKS on RIBS, in the presence of CAMI-T/S, was non-significant (*b* = 0.034, s.e. = 0.019, *P* = 0.081), indicating potential full mediation. Moreover, the indirect effect of MAKS on RIBS via CAMI-T/S was found to be significant (*b* = 0.087, s.e. = 0.010, 95% CI: [0.070, 0.107]), suggesting that CAMI-T/S may fully mediate the relationship between MAKS and RIBS. In other words, all dimensions of an individual’s knowledge about mental health issues, including their ability to recognise common mental disorders, seek support and understand the deleterious effects of mental health stigma, are involved in shaping an individual’s attitudes and subsequent behavioural intentions towards people with mental illness in the community.

**Table 4:** Direct, indirect and total effects of 12-item stigma-related mental health knowledge on intended behaviours via attitudes towards mental illness

Path	*b* ^a^	s.e.	*t*	Bootstrap 95% CI^b^	
				Lower	Upper
(a) Direct effects					
MAKS → CAMI-T/S	0.218	0.017	12.929**	0.185	0.251
CAMI-T/S → RIBS	0.401	0.025	16.394**	0.353	0.449
MAKS → RIBS	0.034	0.019	1.746 (*P* = 0.081)	−0.004	0.071
(b) Indirect effects					
MAKS → CAMI-T/S → RIBS	0.087	0.001	–	0.070	0.107

MAKS, Mental Health Knowledge Schedule; CAMI-T/S, Community Attitudes towards the Mentally Ill, Tolerance and Support subscale; RIBS, Reported and Intended Behaviour Scale.

a. *b* indicates unstandardised coefficients.

b. Bootstrap sample size = 5000.

**P < 0.001.

## Discussion

This article reports findings based on two independent cross-sectional surveys on stigma outcomes among members of the Hong Kong general public, conducted in 2021 and 2023. Overall, the results demonstrated that stigma-related outcomes including stigma-related mental health knowledge (MAKS), attitudes (CAMI) and behavioural intentions (RIBS) worsened between 2021 and 2023. Moreover, individuals who disclosed their mental illness experienced significantly higher levels of well-being; however, the rate of disclosure dropped over the 2 years. In addition, mediation analyses suggested distinct pathways through which mental health knowledge influences behavioural intentions, highlighting the crucial role of comprehensive mental health knowledge in shaping individuals’ attitudes and, consequently, enhancing their behavioural intentions towards those with mental illness.

The worsening of these stigma-related outcomes between 2021 and 2023 can be interpreted in the context of two major events occurring at local and global levels. First, 2021 represents a timeframe within which concerns and public health measures related to the pandemic were in full force. Of note, Hong Kong experienced some of the strictest isolation and quarantine measures in the world, the negative mental health implications of which have been clearly documented in the literature.^4,38,39^ Research on the relationship between COVID-19 and levels of stigma has also identified a rise in general (non-mental-health-related) stigma following the pandemic, which many researchers have attributed to heightened levels of wariness surrounding contamination and sickness resulting from rigid social distancing measures implemented globally.^40,41^ Against this backdrop of increased generalised apprehension, studies have also reported burgeoning levels of mental health stigma.^2,21^

This exacerbation of stigma-related outcomes between 2021 and 2023 can also be viewed in light of a significant mental-health-related event that occurred in close temporal proximity to the administration of the second survey in 2023. Colloquially known as the ‘double stabbing’ event,^42^ the highly public murder of two women in a Hong Kong shopping mall in June 2023 by an individual who reportedly had a history of mental illness led to a sharp increase in negative media portrayals of individuals with mental illnesses. In addition to the immediate online proliferation of video footage surrounding the event, local media reporting on the event emphasised the assailant’s history of mental illness and his continued need for regular psychiatric consultations.^42^ Consistent with the existing literature, representations of mental illness in the media could influence public perceptions of and attitudes towards individuals with mental illnesses in the community. In particular, stigma research has consistently demonstrated that exposure to negative media portrayals of individuals with mental illnesses is associated with increases in misconceptions and stigmatising attitudes towards these individuals.^43–45^

Although this is not a novel finding (e.g. Stratton et al, 2019)^46^, our analysis also demonstrated that despite disclosure being associated with higher well-being outcomes, the proportion of participants willing to disclose their mental health diagnosis decreased between 2021 and 2023 (Table 1).^a^ This is noteworthy, as it demonstrates the costly impact of mental health stigma at not just the societal level but also the individual level. Specifically, this suggests that rising levels of public mental health stigma powerfully influence individual behaviour, leading to shame and possible reluctance to seek social support. This is consistent with the findings of previous studies that have demonstrated how mental health stigma serves to delay or completely inhibit help-seeking behaviours among individuals experiencing various forms and degrees of mental distress.^47–49^

In alignment with the existing literature,^10^ we found that lower prejudicial attitudes towards individuals with mental illness and higher knowledge about mental health topics were correlated with more accommodating behavioural intentions towards people with mental illness. Specifically, our mediational analysis finding indicates that fostering both stigma-related mental health knowledge and supportive attitudes can help to reduce discrimination and promote inclusivity. This emphasises that the different facets of mental health stigma, including knowledge, attitudes and behaviours, must all be considered within mental health anti-stigma interventions. In particular, the idea that mental health stigma is a multifaceted phenomenon that requires multi-pronged intervention approaches by supported in the current research.^9,10,50^

Our study also underscores the importance of considering all dimensions of mental health knowledge when determining the relationships between stigma-related mental health knowledge, attitudes and behavioural intentions. Specifically, our analyses revealed that CAMI-T/S scores partially mediate the relationship between MAKS-A and RIBS scores, whereas CAMI-T/S scores fully mediate the relationship between MAKS (combined score) and RIBS. In other words, it is only when all dimensions of mental health knowledge – including the ability to recognise common mental disorders, identify sources of mental health support and understand the harmful consequences of mental health stigma on the individual – that the relationship between knowledge, attitudes and behavioural intentions can be fully explained. Notably, many mental health programmes in Hong Kong to date have focused on delivering psychoeducation,^51^ which may increase theoretical understandings of mental health issues (e.g. how to recognise common mental disorders) but overlook other crucial facets of mental health knowledge. Importantly, these other facets of mental health knowledge, such as an understanding of how mental health stigma exerts harmful influences on the lives of individuals with mental illness in the community, have prominent roles in shaping stigma-related attitudes and behavioural intentions.^9,52^

Last, it is important to note that this is the first population-wide mental health stigma study in Hong Kong to investigate changes in measures of mental health stigma. The lack of population-wide mental health stigma measures in Hong Kong represents a significant gap in our basic understanding of the level of stigma in the general population. This gap hampers efforts to create informed, effective and equitable responses to address and reduce stigma.

### Implications for future research and practice

Taken together, our findings have several implications for future mental health research and practice. First, they suggest the importance of considering all dimensions of mental health knowledge when designing interventions to address stigmatising attitudes and behavioural intentions towards individuals with mental illness. That is, in addition to increasing participants’ theoretical mental health knowledge (e.g. their ability to recognise common mental disorders), these interventions must aim to enhance participants’ applied knowledge about mental health issues (e.g. when and where to seek help for mental distress, and how mental health stigma can negatively influence the lives of real individuals with mental illness in the community).

Thus, our findings bolster the existing literature suggesting that effective mental health anti-stigma interventions must address stigma with comprehensive consideration of its multiple facets, namely, mental health knowledge, attitudes and behaviours.^50,53^ Second, our finding that willingness to disclose a mental health diagnosis decreased between 2021 and 2023 indicates that anti-stigma interventions should place greater emphasis on providing practical help-seeking strategies. This could include, for instance, offering suggestions for affordable mental health support resources that cater to populations of diverse backgrounds and needs (e.g. individuals with different diagnoses, geographic locations and socioeconomic statuses) and guidelines for how to communicate about one’s mental health diagnosis (e.g. how to start a conversation with a loved one or doctor about requiring more support). Future research should also examine factors affecting disclosure willingness in Hong Kong following the pandemic, as this could serve to inform the development of interventions encouraging proactive help-seeking behaviours.

### Strengths and limitations of the current study

This study had numerous strengths. First, both the survey conducted in 2021 and the one conducted in 2023 had representative sample sizes of more than 1000 respondents, which enhanced the generalisability of our findings. Second, we incorporated validated stigma measures that are used in international studies,^54^ which served to enhance the validity and reliability of our findings. We also examined both combined MAKS scores and scores from the first six MAKS items (MAKS-A) in our analysis. This dual approach provided a robust and comprehensive understanding of the data. Furthermore, it enabled us to address the inconsistencies in previous research and offer a more standardised method for future studies. Finally, this study was conducted during a time when mental health issues were particularly topical in Hong Kong.^4,38^ The findings of this study are therefore timely contributions to the development and enhancement of mental health resources and interventions during this time, and to the literature on mental health stigma in Hong Kong during and after the COVID-19 pandemic.

Limitations of this study include the fact that all findings presented here were quantitative in nature. Although our findings illustrated broad trends in respondents’ knowledge, attitudes and behavioural intentions surrounding mental illness, a deeper investigation of the reasoning behind these trends with an analysis of qualitative data (e.g. short-answer questions and/or semi-structured interviews) would further strengthen them. In addition, as we drew on findings from two serial surveys adopting a cross-sectional design (i.e. the participants in the second sample were different from those in the first), there may have been individual-level differences in stigma outcomes between the two samples. However, it should be noted that as we used random sampling procedures to mitigate any potential selection bias, both samples were broadly representative of the Hong Kong population at the time of data collection.

## Data Availability

The data that support the findings of this study are available on request from the corresponding author, O.T. The data are not publicly available owing to their containing information that could compromise the privacy of research participants.

## Footnotes

a. The WHO-5 measure of well-being was only included in the 2023 survey. Thus, we were only able to report on the findings from our regression analysis conducted on the relationship between mental illness disclosure willingness and well-being outcomes from our 2023 survey data.
